# The occurrence of delayed neuropsychologic sequelae in acute carbon monoxide poisoning patients after treatment with hyperbaric or normobaric oxygen therapy

**DOI:** 10.1097/MD.0000000000024183

**Published:** 2021-01-15

**Authors:** Chih-Chieh Yang, Yi-Fei Chuang, Pei-En Chen, Ping Tao, Tao-Hsin Tung, Ching-Wen Chien

**Affiliations:** a Department of Business Administration, Ming Chuan University, Taipei; b Department of Critical Care Medicine, Lotung Poh-Ai Hospital, Yilan; c Institute of Health Policy and Management, National Taiwan University; d Taiwan Association of Health Industry Management and Development, Taipei; e Division of Medical fees, Department of Medical Affair Administration, Kaohsiung Veterans General Hospital, Kaohsiung; f Enze Medical Research Center, Affiliated Taizhou Hospital of Wenzhou Medical College, Taizhou, Zhejiang,; g Cheng-Hsin General Hospital, Taipei, Taiwan; h Institute for Hospital Management, Tsing Hua University, Shenzhen Campus, China.

**Keywords:** carbon monoxide poisoning, delayed neuropsychiatric sequelae, hyperbaric oxygen, normobaric oxygen

## Abstract

This study aimed at assessing which one of the 2 therapies is better for treating carbon monoxide (CO) poisoning from the perspective of reducing delayed neuropsychologic sequelae (DNS).

We used Taiwan's National Health Insurance Research Database (NHIRD) to conduct a nationwide population-based cohort study to assess which therapy is better for CO poisoning patients. To accurately identify patients with DNS, the definition of DNS is included neurological sequelae, and cognitive and psychological sequele. The independent variable was therapy and the dependent variable was DNS occurred within 1 year after discharge from a medical institution. The control variables were age, gender, the severity of CO poisoning, and comorbidities present before CO poisoning admission.

The risk of developing DNS in patients treated with Hyperbaric Oxygen (HBO) was 1.87-fold (*P* < .001) than normobaric oxygen (NBO) therapy. The severity of CO poisoning and comorbidities were also found to have significant influences on the risk of developing DNS.

HBO may be a risk therapy for treating CO poisoning.

## Abstract 2

Supplemental Digital Content is available in the text

**Highlight**

**What is known about the topic?**

- Acute carbon monoxide (CO) poisoning is a common cause of accidental death and suicides.
- CO poisoning not only harms human health but is also damaging to the brain.
- Normobaric oxygen (NBO) and hyperbaric oxygen (HBO) is commonly used to treat CO poisoning.

**What does this paper add?**

- This study aimed at assessing which one of the two therapies is better for treating CO poisoning from the perspective of reducing DNS.
- CO poisoning patients who were severely poisoned or having comorbidities, such as peptic ulcer disease excluding bleeding, depression, were more likely to occur DNS.
- CO poisoning patients who were treated by HBO had higher outpatient and hospitalization expenditure in one year after CO poisoning than those treated by NHO.

## 1 Introduction

Acute carbon monoxide (CO) poisoning is a common cause of accidental death and suicides. A CO concentration of 5000 ppm or more is typically sufficient to kill a person within a few minutes. However, the incidence of CO poisoning is significantly high worldwide, even in economically developed countries, such as the United States (US). According to the National Vital Statistics System, unintentional and non–fire-related CO poisoning is 2244 deaths annually from 2010–2015.^[1]^ These statistics demonstrate the substantial danger presented by CO poisoning.

CO poisoning not only harms human health but is also damaging to the brain. The delayed neuropsychiatric sequelae (DNS) that commonly occur after treating CO poisoning patients provide the best evidence of this. Previous studies have reported rates between 2% and 30% of CO poisoning survivors who experience DNS after discharge from medical facilities.^[2–4]^ Two therapies are commonly used to treat CO poisoning: normobaric oxygen (NBO) and hyperbaric oxygen (HBO). Because the administration of HBO is faster than NBO and has greater first-aid effectiveness for serious acute CO poisoning, HBO has gradually replaced NBO in many countries.^[5–8]^ However, the cause of the DNS experienced by CO poisoning survivors has not been determined. Some studies have suspected that therapy with either whether HBO or NBO may be one of the causes of DNS is still controversial,^[8–11]^ however, studies aimed at assessing which of the 2 therapies is better for the therapy of CO poisoning, from the perspective of reducing complications, will provide greater clinical benefit to the existing therapy of CO poisoning.

Because the association between the 2 therapies and the occurrence of DNS in CO poisoning survivors has not been firmly established and studies of similar topics conducted using large samples are rare, this study used a population-based database, the Taiwan National Health Insurance Research Database (NHIRD), to assess differences in the risk of DNS in acute CO poisoning survivors who were discharged from medical institutions after receiving HBO or NBO therapy. Factors influencing the time of DNS occurrence were also studied.

## 2 Methods

### 2.1 Data and subjects

This study included that all beneficiaries were entitled to receive National Health Insurance (NHI) services in 2006 to 2009 and the data included all the population. The NHIRD collects every claim record, including reasons of use (5 International Classification of Disease, 9th version, Clinical Modification (ICD-9-CM) diagnostic and operation codes), therapy, medications, and examinations of every NHI beneficiary, which includes more than 99% of Taiwan's total population annually. The NHIRD data are validated by the National Health Research Institute (NHRI) and all identifying information is encrypted before released to the public for research purposes.^[12]^ Therefore, this study was approved by the Institutional Review Board (IRB) in Lotung Poh-Ai Hospital, Yilan, Taiwan (ID: AEP2010120054). All subjects in this study were survivors of acute CO poisoning (ICD-9-CM codes: 986, E868.3, E868.8, E868.9, and E982.1) who received either NBO or HBO therapy in 2007 and 2008. Due to this study set is aiming to whether HBO or NBO is better for treating CO poisoning from the perspective of reducing DNS, patients with DNS before CO poisoning, defined as having DNS diagnoses twice in 2006 were excluded.

### 2.2 Study variables

The independent variable in this study was the treatment method for CO poisoning: HBO or NBO. The dependent variable was whether DNS occurred within 1 year (or 12 consecutive months) after discharge from a medical institution, typically a hospital. DNS was defined as the presence of neurological sequelae, and cognitive and psychological sequelae;^[4,13]^ the details are shown in Appendix 1, http://links.lww.com/MD/F527. The control variables were age, gender, the severity of CO poisoning, and comorbidities present before CO poisoning admission. The period of study subjects follow-up was the time from the index data to the data of the first diagnosis in the inpatient or outpatient. The severity of CO poisoning was determined by the patients therapy during his/her admission to a medical institution, typically a hospital. The severity of CO poisoning was defined as mild if a patient was discharged immediately after an outpatient or emergency room visit and possible symptoms include headache, nausea, vomiting, Vertigo, blurred vision. If the patient was hospitalized and with symptoms such as conscious disturbance, Syncope, chest pain, dyspnea, malaise, tachycardia, tachypnea, the severity of CO poisoning was defined as moderate. If the patient stayed in the intensive care unit (ICU) for more than 1 day during hospitalization and with the situation like palpitation, arrhythmia, hypotension, myocardial ischemia, asystole, apnoea, acute respiratory distress syndrome, Seizures, coma, the severity of CO poisoning was defined as severe. (Appendix 2, http://links.lww.com/MD/F528) Comorbidities were measured according to the Elixhauser comorbidities definition.^[14,15]^ The Elixhauser comorbidity measure developed a list of 30 frequently occurring comorbidities that may affect the outcomes (length of hospital stay, hospital transfer, or mortality) of medical care. We used the Elixhauser comorbidities to assess the impact of each comorbidity on the occurrence of DNS in CO poisoning patients within 1 year after discharge from a medical institution. In addition, to minimize the potential misclassification of the diagnoses each comorbidity was identified by regarding records of the diagnosis on at least 2 occasions (wither outpatient visits or hospital admissions).

### 2.3 Statistical analysis

SAS 9.2 (SAS Institute Inc., Cary, NC, U.S.) was used to perform the statistical analysis. For univariate statistical analyses, the χ^2^ test and *t*-test were used to compare differences between 2 patient groups (receiving HBO or NBO) in DNS occurrence, gender, age, Elixhauser comorbidities, and severity of CO poisoning. Stepwise logistic regression was used to analyze the odds ratio of HBO and NBO therapy to the DNS occurrence in acute CO poisoning patients within 1 year after discharge. The occurrence of DNS within1 year after discharge was analyzed using a Kaplan–Meier curve to determine whether the lengths of time before the occurrence of DNS following the2 therapies were identical. A Cox proportional-hazard regression was performed to investigate the influence of different therapies and control variables on the time to develop DNS. Any variable with fewer than 5 subjects was excluded from the logistic and Cox proportional-hazard regression analyses. A *P* value <.05 was regarded as statistically significant.

In addition, we checked whether the Cox model met the proportionality assumption on the basis of Schoenfeld residuals. Due to if more severe patients in one treatment group died than the other group, then more mild/moderate patients are likely to be left in the treatment group with high mortality rate, which might lead to more desirable outcomes. To avoid the impact of death competing with selection bias, we checked the competitive risk model by considering all competitive causes of death.

## 3 Results

The demographic data of the patients who received therapy for CO poisoning are shown in Table 1. The NBO group accounted for 71.56% (1434 patients) of the 2004 included patients, and the HBO group accounted for the remaining 28.44% (570 patients). The severity level of CO poisoning and the occurrence of solid tumors without metastasis significantly differed between groups. Patients with a moderate level of CO poisoning were the most likely to receive HBO therapy, followed by severe and mild levels of CO poisoning (32.03%, 27.25%, and 26.27%, respectively). The proportion of patients who received NBO therapy and experienced solid tumors without metastasis (2.30%) was higher than that of the patients who received HBO therapy (0.70%).

**Table 1:** Description of subjects by hyperbaric oxygen (HBO) or normobaric oxygen (NBO) treatments.

Variable	Total	NBO	HBO	*P*
Total^∗^	2004 (100.00%)	1434 (71.56%)	570 (28.44%)	
Gender^∗^				.068
Female	933 (49.55%)	729 (50.84%)	264 (46.32%)	
Male	1011 (50.45%)	705 (49.16%)	306 (53.68%)	
Age^†^	33.08 (16.12)	32.95 (16.54)	33.41 (15.02)	.565
Severity of CO poisoning^∗^				.033
Mild	944 (47.11%)	696 (73.73%)	248 (26.27%)	
Moderate	693 (34.58%)	471 (67.97%)	222 (32.03%)	
Severe	367 (18.31%)	267 (72.75%)	100 (27.25%)	
Elixhauser comorbidities^∗^				
Cardiac Arrhythmia				.593
No	1956 (97.60%)	1398 (97.49%)	558 (97.89%)	
Yes	48 (2.40%)	36 (2.51%)	12 (2.11%)	
Hypertension				.071
No	1874 (93.51%)	1332 (92.89%)	542 (95.09%)	
Yes	130 (6.49%)	102 (7.11%)	28 (4.91%)	
Chronic Pulmonary Disease				.349
No	1909 (95.26%)	1362 (94.98%)	547 (95.96%)	
Yes	95 (4.74%)	72 (5.02%)	23 (4.04%)	
Diabetes				.988
No	1937 (96.66%)	1386 (96.65%)	551 (96.67%)	
Yes	67 (3.34%)	48 (3.35%)	19 (3.33%)	
Liver Disease				.573
No	1934 (96.51%)	1386 (96.65%)	548 (96.14%)	
Yes	70 (3.49%)	48 (3.35%)	22 (3.86%)	
Peptic ulcer disease develop bleeding				.115
No	1914 (95.51%)	1363 (95.05%)	551 (96.67%)	
Yes	90 (4.49%)	71 (4.95%)	19 (3.33%)	
Solid Tumor without Metastasis				.016
No	1967 (98.15%)	1401 (97.70%)	566 (99.30%)	
Yes	37 (1.85%)	33 (2.30%)	4 (0.70%)	
Rheumatoid Arthritis/collagen				.863
No	1978 (98.70%)	1415 (98.68%)	563 (98.77%)	
Yes	26 (1.30%)	19 (1.32%)	7 (1.23%)	
Fluid and Electrolyte Disorders				.593
No	1980 (98.80%)	1418 (98.88%)	562 (98.60%)	
Yes	24 (1.20%)	16 (1.23%)	8 (1.40%)	
Alcohol Abuse				.550
No	1982 (98.90%)	1417 (98.81%)	565 (99.12%)	
Yes	22 (1.10%)	17 (1.19%)	5 (0.88%)	
Depression				.791
No	1978 (98.70%)	1416 (98.74%)	562 (98.74%)	
Yes	26 (1.30%)	18 (1.26%)	18 (1.26%)	

∗ Chi-Squared test.

† *t*-test. Congestive Heart Failure, Valvular Disease, Pulmonary Circulation Disorders, Peripheral Vascular Disorders, Paralysis, Other Neurological Disorders, Hypothyroidism, Renal Failure, AIDS/HIV, Lymphoma, Metastatic Cancer, Coagulopathy, Obesity, Weight Loss, Blood Loss Anemia, Deficiency Anemia, Drug Abuse and Psychoses were excluded from further analyses due to not enough patients.

Table 2 shows the distribution of DNS occurrence 1 year after discharge. Within 1 year, 398 patients (19.86%) developed DNS. Significant differences in the occurrence of DNS were observed between therapies and CO poisoning severities, and the presence of comorbidities, such as depression and peptic ulcer disease without bleeding, also significantly increased the risk of DNS. The proportion of patients who received HBO therapy and then developed DNS (27.02%) was higher than that of the patients who received NBO therapy (17.02%). Additionally, more patients with severe CO poisoning developed DNS (28.61%) compared to patients with mild or moderate poisoning (16.95% and 19.19%, respectively). The proportion of patients with peptic ulcer disease without bleeding who developed DNS (30.00%) was higher than that of patients without peptic ulcer disease (19.38%). Similarly, more patients with depression developed DNS (50.00%) than those without depression (19.46%).

**Table 2:** Descriptions of subjects by the occurrence of delayed neuropsychologic sequelae (DNS) 1 year after carbon monoxide (CO) poisoning.

	DNS		
Variable	No	Yes	*P*
Total^∗^	1606 (80.14%)	398 (19.86%)	
Treatment^∗^			<.001
NBO	1190 (82.98%)	244 (17.02%)	
HBO	416 (72.98%)	154 (27.02%)	
Gender^∗^			.841
Female	794 (79.96%)	199 (20.04%)	
Male	812 (80.32%)	199 (19.68%)	
Age^†^	32.75 (16.34)	34.44 (15.05)	.061
Severity of CO poisoning^∗^			<.001
Mild	784 (83.05%)	160 (16.95%)	
Moderate	560 (80.81%)	133 (19.19%)	
Severe	262 (71.39%)	105 (28.61%)	
Elixhauser comorbidities^∗^			
Cardiac Arrhythmia			.864
No	1568 (80.16%)	388 (19.84%)	
Yes	38 (79.17%)	10 (2.51%)	
Hypertension			.239
No	1507 (80.42%)	367 (19.58%)	
Yes	99 (76.15%)	31 (23.85%)	
Chronic Pulmonary Disease			.106
No	1536 (80.46%)	373 (19.54%)	
Yes	70 (73.68%)	25 (26.32%)	
Diabetes			.924
No	1552 (80.12%)	385 (19.88%)	
Yes	54 (80.60%)	13 (19.40%)	
Liver Disease			.783
No	1551 (80.20%)	383 (19.80%)	
Yes	55 (78.57%)	15 (21.43%)	
Peptic ulcer disease develop bleeding			.014
No	1543 (80.62%)	371 (19.38%)	
Yes	63 (70.00%)	27 (30.00%)	
Solid Tumor without Metastasis			.129
No	1580 (80.33%)	387 (19.67%)	
Yes	26 (70.27%)	11 (29.73%)	
Rheumatoid Arthritis/collagen			.936
No	1585 (80.13%)	393 (19.87%)	
Yes	21 (80.77%)	5 (19.23%)	
Depression			.001
No	1593 (80.54%)	385 (19.46%)	
Yes	13 (50.00%)	13 (50.00%)	

∗ Chi-Squared test.

† *t*-test. Congestive Heart Failure, Valvular Disease, Pulmonary Circulation Disorders, Peripheral Vascular Disorders, Paralysis, Other Neurological Disorders, Hypothyroidism, Renal Failure, AIDS/HIV, Lymphoma, Metastatic Cancer, Coagulopathy, Obesity, Weight Loss, Fluid and Electrolyte Disorders, Blood Loss Anemia, Deficiency Anemia, Alcohol Abuse, Drug Abuse and Psychoses were excluded from further analyses due to not enough patients.

As shown in Table 3, after controlling for patients characteristics, CO poisoning severity, and comorbidities, significant differences in the occurrence of DNS existed between the 2 therapy methods. The risk of developing DNS in patients treated with HBO was 1.87-fold greater (*P* < .001) than that of patients who received NBO therapy. In addition, the severity of CO poisoning and comorbidities, including other neurological disorders, peptic ulcer disease without bleeding, and depression, were also found to have a significant influence on the risk of developing DNS. Patients with severe CO poisoning were 1.94-fold (*P* < .01) more likely to develop DNS than patients with mild poisoning. Additionally, the risks of developing DNS were 4.04- (*P* = .038), 1.83- (*P* = .012), and 3.46- (*P* = .003) fold greater in patients with other neurological disorders, peptic ulcer disease without bleeding and depression than that for patients without these comorbidities.

**Table 3:** A stepwise logistic regression on the occurrence of delayed neuropsychologic sequelae (DNS) for carbon monoxide (CO) poisoning patients after different treatments.

Variable	$\overset{\circ }{\beta }$	SE ($\overset{\circ }{\beta }$)	Adjusted OR	*P*
Treatment				
NBO (ref)				
HBO	0.62	0.12	1.87	<.001
Severity of CO poisoning				
Mild (ref)				
Moderate	0.12	0.13	1.12	.376
Severe	0.66	0.15	1.94	<.001
Elixhauser comorbidities				
Other Neurological Disorders				
No (ref)				
Yes	1.39	0..67	4.04	.038
Peptic ulcer disease develop bleeding				
No (ref)				
Yes	0.61	0.24	1.83	.012
Depression				
No (ref)				
Yes	1.24	0.41	3.46	.003

ref = reference group.

**Figure 1:**
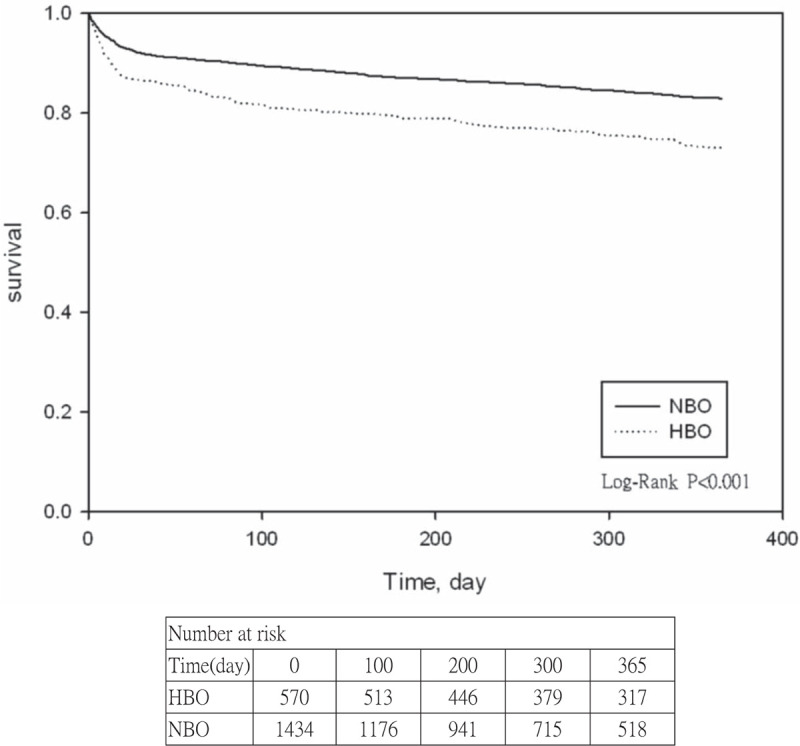
Kaplan–Meier curve of therapies of carbon monoxide (CO) poisoning and delayed neuropsychologic sequelae (DNS).

Figure 1 shows the descriptive Kaplan–Meier curve for DNS occurrence and the time of occurrence within 1 year after poisoning. This curve indicates that the time between discharge following HBO therapy and the occurrence of DNS was longer than the time following NBO therapy and DNS occurrence (log-rank *P* < .0001). As shown in Table 4, the Cox model met the proportionality assumption (*P* = .09). After controlling for gender, age, Elixhauser comorbidities, and CO poisoning severity, the Cox proportional-hazards regression identified significant differences among therapy methods, CO poisoning severity, and the presence or absence of comorbidities, including peptic ulcer disease without bleeding and depression. The hazard ratio of patients who received HBO therapy and then developed DNS was 1.87-fold greater than that of patients who received NBO. The DNS risk for patients with severe CO poisoning was 1.63-fold greater than that for patients with mild poisoning. The risks of developing DNS among patients with peptic ulcer disease without bleeding and those with depression were 1.61 and 2.07-fold greater than in patients without these comorbidities.

**Table 4:** Cox proportional-hazards regression.

Varible	$\overset{\circ }{\beta }$	SE ($\overset{\circ }{\beta }$)	Hazard Ratio	*P*
Treatment				
NBO (ref)				
HBO	.62	0.11	1.87	<.001
Gender				
Female (ref)				
Male	−.16	0.11	0.85	.132
Age	.01	0.01	1.01	.373
Severity of CO poisoning				
Mild (ref)				
Moderate	.09	0.13	1.09	.487
Severe	.49	0.14	1.63	.001
Elixhauser comorbidities				
Cardiac Arrhythmia				
No (ref)				
Yes	−.01	0.36	0.99	.998
Hypertension				
No (ref)				
Yes	.29	0.23	1.33	.215
Chronic Pulmonary Disease				
No (ref)				
Yes	.30	0.23	1.35	.185
Diabetes				
No (ref)				
Yes	−.38	0.33	0.68	.256
Liver disease				
No (ref)				
Yes	−.06	0.29	0.94	.833
peptic ulcer disease excluding bleeding				
No (ref)				
Yes	0.48	0.22	1.61	.027
Solid Tumor without Metastasis				
No (ref)				
Yes	0.27	0.35	1.31	.448
Rheumatoid arthritis/collagen				
No (ref)				
Yes	−0.29	0.51	0.75	.572
depression				
No (ref)				
Yes	0.72	0.32	2.07	.024

ref = reference group.

## 4 Discussions

### 4.1 Clinical implications

Table 5 presents the previous studies on DNS following CO poisoning.^[4,9,6–18]^ However, we could not state which therapy is better owing to the disparity of the included studies. The results of this study indicate that the risk of developing DNS within 1 year after hospital discharge in CO poisoning patients was higher in patients treated with HBO compared to those treated with NBO. We used both stepwise logistic regression and Cox regression to adjust cofounding factors and minimize the impacts on the study. These results can be further discussed from 3 perspectives.

**Table 5:** The different treatments for acute myocardial infarction patients in various populations.

First author	Study year	Study design	Screened number	Setting	Outcomes	Conclusion	Reference
Thom et al	1995	Prospective randomized	60	USA	DNS: 0/30 (HBO) vs 7/30 (ambient pressure 100% oxygen) (*P* < .05)	DNS after CO poisoning cannot be predicted on the basis of a patient's clinical history or CO level. HBO treatment decreased the incidence of DNS after CO poisoning.	^[16]^
Scheinkestel et al	1999	Randomised controlled double-blind trial	191	Australia	DNS: 5/104 (HBO) vs 0/87 (NBO) (*P* = .03).	Both groups received high doses of oxygen, HBO therapy did not benefit, and may have worsened, the outcome. We cannot recommend its use in CO poisoning.	^[9]^
Pepe et al	2011	Retrospective	347	Italy	HBO therapy:96; NBO therapy: 25134 (DNS)/141 (patients were accessed at 30 days from discharge)	Treatment algorithms based on an appropriate risk-stratification of patients in the Emergency Department might reduce DNS incidence.	^[4]^
Chang et al	2017	Retrospective	81	Taiwan	DNS: 7/21 (HBO) vs 5/60 (NBO) (*P* = .006)	For those with treatment in the intensive care unit because of prolonged loss of consciousness and rescue by a ventilator, special attention should be given and follow-up should be performed to determine whether DNS or PNS occurs, particularly epilepsy and cognitive deficits.	^[17]^
Lin et al	2018	Meta-Analysis	218 (2 studies)	Taiwan	HBO treated patients have a lower incidence of DNS.RR (95% CI): 0.325 (0.02–5.97)	The meta-analysis indicated that compared with CO poisoning patients treated with NBO, HBO treated patients have a lower incidence of neuropsychological sequelae, including headache, memory impairment, difficulty concentrating, disturbed sleep, and delayed neurological sequelae. Taking into consideration the cost-effectiveness of one session of HBO, one session of HBO treatment could be an economical option for patients with CO poisoning with high severity.	^[18]^
Wang et al	2019	Meta-analysis	3 studies	China	Moderate sequelae rate HBO vs control:(RR: 0.95; 95% CI: 0.78–1.16; *P* = .639)Severe sequelae rate HBO vs. NBO:(RR: 2.15; 95% CI: 0.44–10.40; *P* = .343)	These results indicate that HBO therapy significantly reduces the risk of memory impairment compared to NBO, but 2 sessions of HBO might not be better for memory impairment than 1 session of HBO.	^[19]^

RR = risk ratios.

First, brain injuries or lesions that occur after CO poisoning develop gradually. Several hours may pass before patients reach the ED to receive HBO or NBO therapy. Although HBO can rapidly reduce H_b_CO concentrations (the half-life of H_b_CO under HBO is approximately 26 minutes compared to 1 hour under NBO), it may already be too late to reduce or alleviate damage to the central nervous system.^[19]^ As shown in our study, 19.86% of CO poisoning patients developed DNS within 1 year after discharge. This finding is similar to results reported in previous studies.^[2]^ In contrast to those studies, the CO poisoning levels in our study were not based on clinical data but were instead derived from the therapy requirements at the time of poisoning. The results indicate that patients with severe CO poisoning were 1.94-fold more likely to develop DNS in the following year compared to patients with mild poisoning. After controlling for the influence of CO poisoning severity, we still found that CO poisoning patients who received HBO therapy were 1.87-fold more likely to develop DNS when compared to patients who received NBO therapy.

The potential benefit of HBO therapy for patients with CO poisoning has been controversial for many years. Several studies have contended that HBO can better reduce the occurrence of DNS compared to NBO, whereas other studies have argued that HBO therapy does not necessarily result in superior prognoses.^[2,8–11]^ For example, Gilmer et al.^[11]^ compared the probability of developing DNS following HBO and NBO therapy in mice. The results indicated that HBO therapy did not protect against DNS. Scheinkestel et al^[9]^ found that the performance of patients who received HBO during learning and abnormality tests was inferior to that of patients who had received NBO. After monitoring patients for 1 month, they concluded that a significantly greater number of patients who had received HBO therapy developed DNS when compared to patients who received NBO therapy. Although the results of our study support the findings of Scheinkestel et al (1999) the study has its limitations, especially the delayed HBOT and 3 days continuous 100% oxygen therapy that is not the standard practice. Moreover, the other randomized controlled trials did not show any deleterious effects of HBOT, although 3 of them did not support the efficacy of HBOT in preventing DNS following CO poisoning.^[9–11]^ This indicates that HBO therapy does not provide the expected superior benefits for patients with CO poisoning compared to NBO therapy. As stated in previous studies, HBO therapy cannot improve the prognosis for patients with CO poisoning who are in sustained comas.^[10]^ HBO may even cause oxygen poisoning in certain patients, producing symptoms of epilepsy, which deteriorated their prognosis.^[9]^ Therefore, whether rapid increases in brain oxygen concentrations worsen the symptoms of CO poisoning requires further investigation.

Second, the results of previous studies indicate that the binding force of CO to hemoglobin is 200- to 250-fold greater than that of oxygen to hemoglobin.^[20]^ This reduces the amount of oxygen released into the tissue, causing hypoxia of the tissue cells.^[21]^ Accordingly, the principle of HBO therapy is to use the pressure generated by increasing the dissolved oxygen concentration in the blood to accelerate the ability to remove CO hemoglobin and to enhance the cells ability to repair. However, the results of our analysis contradict this principle. Previous studies have suggested that restrictions in the function of hemoglobin and the left-shifted oxygen-hemoglobin disassociation curve may not be the most important factors for explaining CO pathology.^[11]^ Because the initiation of HBO therapy following the occurrence of CO poisoning is typically delayed, the remaining CO hemoglobin in the blood is unlikely to be the only significant factor in the development of neuropsychiatric sequelae. Other cellular death mechanisms, such as caspase-mediated apoptosis, may be related to DNS caused by CO.^[22,23]^ Some studies have also suggested that this may be why patients who received HBO therapy had a higher risk of developing DNS. Therefore, patients with severe CO poisoning or unstable vital signs are not suitable for admission to the HBO cabinet for 2 to 3 hours. For patients receiving endotracheal intubation and respiratory support, the oxygen concentrations in their respirators should be adjusted to achieve effects that are similar to those of HBO therapy.

Third, previous studies have indicated that HBO therapy can efficiently reduce H_**b**_CO ratios more rapidly than NBO therapy.^[24,25]^ Although HBO is useful and offers the advantage of preventing respiratory failure caused by excessive H_**b**_CO concentrations, HBO does not mitigate other common comorbidities of CO poisoning, such as DNS. Previous therapy concepts tended to prescribe HBO therapy for patients with more severe symptoms (H_**b**_CO 10%–25%) or HbCO > 25% and NBO therapy for patients with milder symptoms.^[26]^ The groupings in this study indicate that HBO does not provide superior DNS therapy for patients with mild, moderate, or severe symptoms and that it even increases DNS occurrence. After analyzing nationwide data, we conclude that the use of HBO therapy for patients with CO poisoning offers no DNS prevention benefits. Additionally, the presence of other neurological disorders, depression, and peptic ulcer disease without bleeding at the time of CO poisoning positively influenced the subsequent development of DNS. From the clinical perspective, the possible pathophysiologic mechanism may that factors leading to peptic ulcer, in addition to drugs, stress hormones produced by life pressure are also a major cause of the peptic ulcer. Stress hormones are secreted by the brain, which may be related to DNS. In Scheinkestel et al. (1995), Hay et al. (2002) both demonstrate there is an association between depression, other neurological disorders, and DNS.^[9,27]^ These findings are worthy of further research, having been rarely discussed in previous studies.

In this study, the proportion of DNS (19.86%) was higher than expected. A possible explanation for this is that the diagnosis of DNS includes both persistent neurological sequelae (PNS) and DNS. According to Ning et al (2020) and Lin et al (2018), persistent neurological sequelae (PNS) is a direct result of hypoxic brain damage, which is defined as the neurological symptoms evident at the presentation that persist throughout hospitalization. By contrast, DNS frequently occurs within a few weeks after initial clinical recovery from acute CO poisoning, and its incidence could range from 3% to 40%.^[28,29]^ There are many PNS common clinical symptoms such as seizures, severe encephalopathy, or coma. These patients are usually needed to maintain a follow-up procedure in neurologic clinics after the discharge. On the contrary, there are also other common clinical symptoms of DNS on some specific group of patients such as insomnia, anxiety; and these patients usually have to carry the follow-up treatments in the psychiatric outpatient clinic. We used the NHIRD to identify the ICD- 9 cm code of DNS instead of PNS, which can exclude most patients with only PNS but cannot exclude patients with both PNS and DNS.

This study differs from previous research in that the majority of previous studies were either small-sample studies examining a single or multiple medical units or involved clinical experiments on animals. By contrast, this study was a population-based study, using nationwide data to compare the comorbidities and severity of CO poisoning patients who received HBO or NBO therapy. Thus, selection bias was less likely to occur, and the results of this study were more representative.

### 4.2 Methodological considerations

This study had several limitations. First, it used data from the health care administrative database for statistical analysis. Although the NHIRD lacked information on CO poisoning severity (CO hemoglobin concentration), we used the degree of hospitalization to define the severity of CO poisoning. Besides, we also included the gender, age, and Elixhauser comorbidities of CO poisoning patients as control variables. These variables might correct errors between patients with differing severities. We expected their inclusion to reduce the variation between the2 groups and thus increase the accuracy of the results. Second, the NHIRD lacked clinical data on CO hemoglobin concentrations. Second, we were unable to obtain the length of time patients may have been exposed to CO or the duration between the discovery of CO poisoning and therapy from the NHIRD. We also had no formation of concomitant treatments in addition to HBO or NBO among study patients. These factors may have affected the incidence of DNS among patients receiving different oxygen therapies. In addition, to reduce potential bias due to confounding and informative censoring, further study should further adjust for not only different levels of hospitalization, but also loss of consciousness is a necessary prognosis that may affect both treatment assignment and outcome. Types of clinical facilities where subjects were admitted to also should be accounted for given the fact that preference and availability of treatments vary among clinical settings, which in turn might relate to outcome. Third, several potential confounders initial consciousness and COHb level, the time elapsed before emergency department visit, and co-exposure to other toxicants, and the inherent limitations of using an NHIRD database (such as coding errors), the lack of detailed clinical data, also the higher risk of DNS among patients receiving HBOT are likely to be due to confounding by indication. Such us patients with more severe CO poisoning are more likely to receive HBOT then those with less severe effects of CO poisoning. Fourth, this study used admission or ICU admission as a surrogate indicator for the severity of CO poisoning. However, the classification of severity is likely to be inaccurate because the indication for hospital or ICU admission varies widely between hospitals in Taiwan. In the lack of detailed clinical data, this will result in residual confounding even though “the severity of poisoning” has been controlled in the analysis. Fifth, Base on the overall population in NHIRD we used in this study, the internal and external validity is acceptable. However, the database employed is relatively old. Sixth, on average, the development of DNS was around 40 days, without extension to one year. As the data was extracted from the NHRID, there might be selection bias in the treatment group as the enrollment of no DNS that received HBO may base on the clinical judgment rather than a random selection. The outcomes may also be biased if the coding of DNS is missed in some patients. Seventh, patients with a moderate level of CO poisoning or severe level received HBO, we already adjust the bias by regression; however, the therapy decision may influence the poor clinical outcome (DNS) based on the clinical judgment bias, we could not completely exclude the possibility of judgement bias. Eighth, in this study, we stated that rapid increases in brain oxygen concentration may worsen the symptoms of CO poisoning. However, this is based on the statistic model rather than a direct prospective measure compared to 2 randomized study arm. Final, generally, the outcomes of HBO in brain recovery depend on the timing of treatment. However, the information we can access not including CO poisoning time and treatment duration (use interval between poisoning and HBO), and onset time.

## 5 Conclusions

In conclusion, HBO may be a risky therapy than NBO for CO poisoning patients because the occurrence of DNS after HBO therapies was significantly frequent than that of NBO. More studies, especially cost and effectiveness studies, should be conducted before a final consensus of how and when to use which therapy for CO poisoning patients can be reached.

## Author contributions

**Conceptualization:** Chih-Chieh Yang.

**Data curation:** Chih-Chieh Yang, Pei-En Chen, Ping Tao, Tao-Hsin Tung.

**Formal analysis:** Tao-Hsin Tung.

**Project administration:** Ping Tao.

**Supervision:** Yi-Fei Chuang, Tao-Hsin Tung, Ching-Wen Chien.

**Visualization:** Pei-En Chen.

**Writing – original draft:** Chih-Chieh Yang, Yi-Fei Chuang.

**Writing – review & editing:** Pei-En Chen, Tao-Hsin Tung.

## Abbreviations

Abbreviations: CO = carbon monoxide, DNS = delayed neuropsychologic sequelae, HBO = hyperbaric oxygen, ICD-9-CM = International Classification of Disease, 9th version, Clinical Modification, ICU = intensive care unit, NBO = normobaric oxygen, NHI = National Health Insurance, NHIRD = Taiwan's National Health Insurance Research Database, PNS = persistent neurological sequelae.
